# GAPS-EUS: a new and reliable tool for the assessment of basic skills and performance in EUS among endosonography trainees

**DOI:** 10.1136/bmjgast-2021-000660

**Published:** 2021-06-09

**Authors:** Per Hedenström, Giovanni Marasco, Leonardo Henry Eusebi, Bjorn Lindkvist, Riadh Sadik

**Affiliations:** 1 Department of Molecular and Clinical Medicine, Institute of Medicine, University of Gothenburg Sahlgrenska Academy, Goteborg, Sweden; 2 Department of Internal Medicine, Sahlgrenska University Hospital, Goteborg, Sweden; 3 Department of Medical and Surgical Sciences, IRCCS Azienda Ospedaliero-Universitaria di Bologna, Bologna, Italy; 4 Department of Medical and Surgical Sciences, University of Bologna, Bologna, Italy

**Keywords:** endoscopic ultrasonography, surgical training, endoscopic procedures, gastrointestinal neoplasia

## Abstract

**Objective:**

Endosonography (EUS) is a useful but complex diagnostic modality which requires advanced endoscopy training and guidance by a supervisor. Since learning curves vary among individuals, assessment of the actual competence among EUS trainees is important.

**Design/methods:**

We designed a novel assessment tool entitled Global Assessment of Performance and Skills in EUS (GAPS-EUS) for assessing skills among EUS trainees. Five quality indicators were marked on a five-grade scale by the supervisor (Observer Score) and by the trainee (Trainee Score). Trainees were included in two high-volume centres (Gothenburg, Sweden, and Bologna, Italy). Outcomes were feasibility, patient safety, reliability, and validity of GAPS-EUS in trainee-performed EUS procedures.

**Results:**

Twenty-two EUS-trainees were assessed in a total of 157 EUS procedures with a completion rate of 157/157 (100 %) and a patient adverse event rate of 2/157 (1.3 %; gastroenteritis n=1, fever n=1). GAPS-EUS showed a high measurement reliability (Cronbach’s alpha coefficient=0.87) and a high inter-rater reliability comparing the supervisor and the trainee (r=0.83, r^2^=0.69, p<0.001). The construct of GAPS-EUS was verified by comparing low-level and high-level performance procedures and the content validity by recording that the EUS-FNA manoeuvre resulted in a lower score than other aspects of EUS 3.07 (95% CI 2.91 to 3.23) vs 3.51 (95% CI 3.37 to 3.65) (p<0.001). External validity was confirmed via similar findings in both centres.

**Conclusion:**

GAPS-EUS is an easy-to-use and reliable tool with a recorded high validity for the assessment of competence among trainees in EUS. It can be recommended to centres involved in the education of future endosonographers.

**Trial registration number:**

NCT02455570.

**Summary box**

**What is already known about this subject?**

- Endosonography (EUS) is regarded as one of the most complex procedures in advanced gastrointestinal endoscopy. Moreover, the learning curves in procedures such as EUS vary significantly among trainees. Consequently, the number of procedures performed during an EUS fellowship is a poor marker for true competence. Therefore, the assessment of trainee skills and performance in a clinical setting is important.

**What are the new findings?**

- We developed and evaluated a new tool (Global Assessment of Performance and Skills in EUS (GAPS-EUS)) for the assessment of skills and performance among trainees in EUS. The tool was proven easy-to-use and patient safe, and it showed a high tool reliability and validity. There is a general lack of such tools with respect to EUS and GAPS-EUS is the first tool properly evaluated in European centres.

**How might it impact on clinical practice in the foreseeable future?**

- The implementation of GAPS-EUS opens up for a systematic evaluation of true skills and performance among EUS trainees in different phases of training. The tool can support supervisors in the arrangement of continued training and facilitate in the estimation of training needed until full competence will be obtained. Thereby, the level of performance in EUS quality indicators can be monitored in all trainees.

## Background

Endosonography (EUS) is a powerful and mini-invasive tool in the diagnostic work-up of suspected malignancy. By use of the echoendoscope, the endosonographer can visualise and assess a wide range of intrathoracic or intrabdominal lesions situated in, or adjacent to, the upper gastrointestinal tract. If needed, lesions can also be targeted and sampled by EUS-guided fine needle aspiration (EUS-FNA) or EUS-guided fine-needle biopsy acquisition (EUS-FNB).^1 2^

Within the field of gastrointestinal endoscopy, EUS is considered among the most challenging procedures from a learning point of view.^3^ Knowledge and competence in EUS, as well as in other endoscopic procedures, encompass both technical and cognitive skills.^4–6^ Typically, technical skills include oesophageal intubation, scope navigation, management of the ultrasound settings, and sampling of target organs. Meanwhile, cognitive skills cover patient risk assessment, interpretation of the ultrasound image, and finally, risk–benefit assessment in EUS-guided interventions. Unsupervised self-learning of EUS is not found to be effective and should be avoided.^7–9^ Instead, the ideal educational environment for high-quality EUS training requires supervision by an experienced and motivated instructor.^9^ Hence, a future EUS trainee, and the appointed supervisor, should plan for a long period of training, and a steep learning curve should not to be expected.

Traditionally, guidelines recommend a minimum of 2 years of basic endoscopy training before starting a trainee fellowship in EUS.^7^ It can be hypothesised that high competence in routine endoscopy will facilitate learning in EUS. However, it is not known if the endoscopy experience of the EUS trainee is associated with fast learning and successful acquisition of EUS skills.^10 11^

Regarding the length of the training period, a minimum of 125 supervised EUS procedures have been suggested in order to achieve reasonable competence in EUS.^7^ However, that number is only a rough estimation, and it does not guarantee that full competence has been obtained in a certain trainee. Lesions with a certain origin and character, such as pancreatic neoplasms, are known to be demanding from a diagnostic point of view.^12 13^ Moreover, linear echoendoscopes have largely replaced radial ones, which make the number of cases in early guidelines unreliable.^9^ In the end, and most importantly, there are considerable differences among trainees in the pace of learning and the time needed to master a complete EUS procedure in all sorts of cases.^3^

Therefore, there is an evident need for a sensitive, but still easy-to-use, assessment tool to be applied on trainees in EUS during different phases of training. The overall aim of the current study was to design, evaluate, and validate a new assessment tool for the evaluation of skills, competence, and learning progress among trainees in EUS.^14^

## Methods

### Global Assessment of Performance and Skills in EUS (GAPS-EUS) assessment tool

We designed a novel assessment tool entitled GAPS-EUS with the purpose to monitor and evaluate trainees in EUS and with respect to competence in all aspects of the EUS procedure (online supplemental materials 1 and 2). The tool was to cover the quality indicators and performance measures suggested by the 5American Society for Gastrointestinal Endoscopy and the European Society of Gastrointestinal Endoscopy,^4^ and its design was also influenced by previous tools suggested for the assessment of endoscopists performing gastroscopy and colonoscopy.^15^ Indeed, similar quality indicators have been used in routine endoscopy.^15^ The first indicator (A) covers skills such as scope handling, intubation, and scope navigation. The second indicator (B) covers trainee competence in the detection and recognition of the ultrasound anatomy and the ability of ultrasound image fine tuning. The third indicator (C) includes the identification and assessment of the ultrasound pathology. If there is indication for EUS-guided sampling during the procedure, the trainee performance of EUS-FNA/EUS-FNB is assessed as the fourth indicator (D). The fifth indicator displays the overall examination quality including patient management (E).

10.1136/bmjgast-2021-000660.supp1 Supplementary data

10.1136/bmjgast-2021-000660.supp2 Supplementary data

All indicators were scored and marked on a five-graded scale, depending on the autonomy and the skills of the trainee. A high-quality procedure independently performed by the trainee was rewarded with a maximum mark of 5, while a low-quality procedure with a need for high-degree verbal guidance and practical assistance by the supervisor was given a minimum mark of 1 (online supplemental material 1). GAPS-EUS was designed to cover both cross-sectional assessments in any trainees and repeated assessments in a single trainee evaluated over time.

### Study setting and design

In a dual-centre tertiary endoscopy unit setting (Gothenburg, Sweden, and Bologna, Italy), trainees in EUS were screened for study inclusion during the time frame 2014–2019. Eligible for inclusion were (1) trainees with an EUS trainee fellowship projected for at least 3 months at one of the two study centres, so called fellowship trainees (FTs), or (2) trainees participating in the annual EUS workshop at the Sahlgrenska University Hospital, so called visiting trainees (VTs). In addition, all trainees being eligible should have a minimum experience of 15 EUS procedures before the first study GAPS-EUS assessment. Trainees were excluded from the study if they were unwilling to participate.

The attending and supervising endosonographers (Gothenburg n=1 (RS), Bologna n=1 (LHE)) responsible for the trainee assessments were both experts in EUS with a minimum of 1500 EUS procedures performed.

All sorts of EUS-guided therapeutic procedures, such as drainage of peripancreatic fluid collections, were excluded from study inclusion as were procedures without an attending EUS supervisor responsible for the trainee assessment. Procedures were also excluded if eligible study patients either presented with an abnormal upper GI anatomy, which made a complete procedure not possible, or had advanced comorbidities making a trainee-performed EUS questionable from an ethical point of view.

The study was registered at ClinicalTrials.gov.

### Trainee-performed EUS procedures and the trainee assessment by GAPS-EUS

Before the first trainee assessment by GAPS-EUS, the background and endoscopy experience of the included trainees were recorded (online supplemental materials 1 and 2). Each VT was evaluated with one cross-sectional spot assessment only. The FTs were evaluated repeatedly with GAPS-EUS in every consecutive trainee-performed EUS.

All study patients were examined by EUS under conscious sedation (midazolam and/or alphentanile) and by the use of a linear echoendoscope (Sahlgrenska: Pentax EG3870UTK (Tokyo, Japan); Bologna: Olympus GF-UCT180 (Pennsylvania, USA)). Present on-site was the trainee to be assessed (hereafter called the *trainee*), the assessing EUS expert (hereafter called the *observer*), and the endoscopy assistants.

First, the trainee was to read the patient referral and decide on the outline of the EUS procedure. Then, the trainee should take responsibility for the patient sedation and start the procedure with routine oesophageal intubation. During the rest of the EUS procedure, the trainee should independently navigate the echoendoscope and carefully display and describe all relevant organs and anatomical structures asked for by the observer. Likewise, all relevant pathology should also be identified and classified by the trainee. If there is an indication for EUS-FNA/EUS-FNB, the first needle pass was performed by the trainee. Any additional needle passes were performed by the observer, who also revised the area of interest before terminating the procedure. All patients examined were monitored post-EUS according to local routines.

After the EUS procedure, the observer entered all data and scores on the trainee performance in the *Observer* version of the GAPS-EUS assessment tool (online supplemental material 1). Simultaneously, and blinded to the scores entered by the observer, the trainee entered the scores on his/her own performance in the trainee version of the GAPS-EUS assessment tool (online supplemental material 2). Finally, immediate feedback on the performance was given to the trainee by the observer.

### Patient follow-up

All patients were subjected to follow-up according to local routines and at least until the final diagnosis was determined. Any adverse event with potential relation to the study procedure was recorded according to the Clavien-Dindo classification.^16^ In addition, the complete medical files of all study subjects were screened by one of the study authors for a minimum of 3 months post-EUS.

### GAPS-EUS calculations

The individual score of the three key indicators (echoendoscope handling (score A), recognition of the ultrasound anatomy (score B), and assessment of the ultrasound pathology (score C)) was recorded together with scores D and E (online supplemental materials 1 and 2). Furthermore, and by using the individual GAPS-EUS indicator scores recorded, a mean observer indicator score was calculated:

Observer Score=(score A+score B+score C+score E)/4.

If EUS-guided sampling was performed, score D was recorded separately.

Likewise, and by use of the individual GAPS-EUS indicator scores recorded in online supplemental material 2, a mean trainee self-assessment indicator score was calculated:

Trainee Score=(score A+score B+score C+score E)/4.

If EUS-guided sampling was performed, score D was recorded separately.

Finally, a mean of the above two scores was calculated:

Compound Score=(Observer Score**+**Trainee Score)/2.

The performance, that is, the quality of the procedure, was ranked accordingly:

- Observer Score: <3 low performance level
- Observer Score: ≥3 –<4 moderate performance level
- Observer Score: ≥4 high performance level

### Study outcomes

Study outcomes were the feasibility, the patient safety, the reliability, and the validity of GAPS-EUS as an assessment tool in trainee-performed EUS procedures.

The feasibility was measured as the completion rate of the assessment tool.

The patient safety was measured as the number of adverse events after a trainee-performed GAPS-EUS procedure.

Three aspects of the reliability of GAPS-EUS were addressed:

First, the internal consistency (measurement reliability) of the GAPS-EUS indicator scores included in Observer Score and Trainee Score was estimated by using Cronbach’s alpha. Crohnbach’s alpha estimates to what extent different components of a certain assessment tool is actually measuring the variable of interest, that is, competence in EUS. Crohnbach’s alpha value ranges between 0 (low consistency) and 1 (high consistency).

Second, the consistency of assessments in between the observer and the trainee (inter-rater reliability) was estimated by calculating the correlation coefficient of the Observer Score and the Trainee Score.

Third, the reliability of GAPS-EUS across the rating scale was depicted as a Bland-Altman plot of the Observer Score and the Trainee Score in all trainee-performed EUS procedures.

Three aspects of the validity of GAPS-EUS were addressed.

The construct validity of GAPS-EUS was evaluated first by recording the performance of FT1 over time, that is, the EUS learning curve. The construct validity was also evaluated by comparing the experience in low performance-level procedures (Observer Score<3) with high performance-level procedures (Observer Score≥4). Finally, the construct validity was evaluated by comparing the Observer Score in different types of lesions with respect to tumour location and tumour character.

The content validity was measured as the capacity of GAPS-EUS in covering multiple aspects of the EUS procedure and that by assessing the trainee performance at EUS-guided sampling and comparing these results with the other parts of the procedure.

The external validity of GAPS-EUS was evaluated by including trainee and patients in two separate training centres—Gothenburg, Sweden, and Bologna, Italy.

### Statistics

Baseline descriptive, continuous data were described as median and IQR, while descriptive, categorical data were described as frequencies. The Observer Score and the Trainee Score were calculated and displayed with the 95% CI.

The correlation of any continuous variables was tested and measured by using Pearson’s correlation coefficient (r).

Student’s t-test was applied in the comparison of the mean scores in between groups.

A Cronbach alpha coefficient of >0.6 was regarded sufficient for acceptable internal consistency and thereby sufficient to accept Observer Score and Trainee Score as Compound Scores of the individual indicator scores.

A p value of <0.05 was considered statistically significant in all analyses.

The statistical calculations and tests were performed using IBM SPSS Statistics V.25.0.

## Results

**Figure 1:**
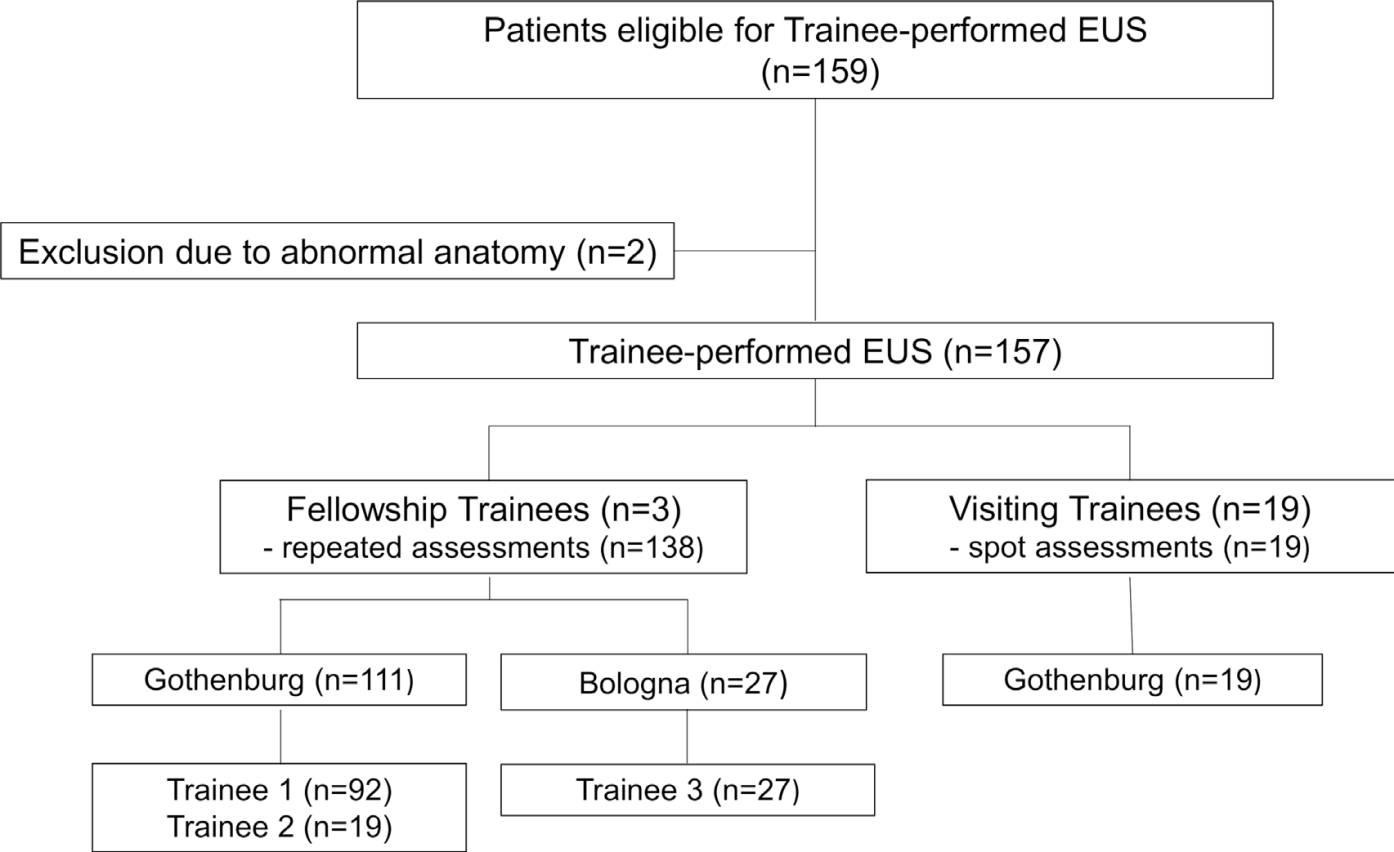
Flowchart of the study enrolment process. EUS, endosonography.

Twenty-two trainees (FT n=3, VT n=19) were evaluated and assessed with the GAPS-EUS tool in a total of 157 procedures (table 1 and figure 1). The median age of the study patients was 64 years (IQR 52–74) and 79/157 (50 %) were women. Further baseline characteristics of the study patients and the lesions examined are presented in table 2. No case was lost from follow-up.

**Table 1:** Characteristics of the study EUS trainees at the time of the first GAPS-EUS assessment

Trainee	Profession	Sex	Age (years)	Gastroscopy (years of practice)	EUS (years of practice)	EUS supervision (n)	EUS independent (n)	EUS all (n)
FT1	Gastroenterologist	M	38	4	<1	50	0	50
FT2	Gastroenterologist	M	39	11	1–2	100	20	120
FT3	Gastroenterologist	M	29	2	<1	30	0	30
VT1	Gastroenterologist	M	51	18	3–5	200	200	400
VT2	Surgeon	M	44	8	<1	10	0	10
VT3	Gastroenterologist	M	53	20	<1	5	0	5
VT4	Gastroenterologist	F	34	4	1–2	10	30	40
VT5	Gastroenterologist	M	50	22	1–2	50	15	65
VT6	Gastroenterologist	F	40	13	3–5	150	150	300
VT7	Gastroenterologist	M	51	25	1–2	50	0	50
VT8	Surgeon	M	50	13	1–2	25	150	175
VT9	Surgeon	M	36	10	1–2	50	0	50
VT10	Surgeon	F	50	10	<1	5	0	5
VT11	Surgeon	F	43	13	<1	5	0	5
VT12	Gastroenterologist	F	45	9	1–2	50	20	70
VT13	Surgeon	F	49	15	1–2	150	40	190
VT14	Gastroenterologist	M	52	18	<1	15	0	15
VT15	Surgeon	M	46	10	1–2	50	60	110
VT16	Surgeon	M	47	9	1–2	50	70	120
VT17	Surgeon	M	51	15	1–2	30	200	230
VT18	Surgeon	M	38	12	1–2	50	10	60
VT19	Surgeon	F	52	12	1–2	20	10	30

EUS, endosonography; F, female; FT, fellowship trainee; GAPS-EUS, Global Assessment of Performance and Skills in EUS; M, male; VT, visiting trainee.

**Table 2:** Patient and lesion characteristics

	All procedures	Procedures by FT	Procedures by VT
Patient characteristics			
Number of patients, n	157	138	19
Patient age (years), median (IQR)	64 (52–74)	65 (51–74)	62 (53–72)
Patient gender, M/F	78/79	73/65	5/14
Lesion characteristics			
Tumour location, n			
Pancreas	79	65	14
Stomach	41	37	4
Paraintestinal	12	12	0
Duodenum	8	7	1
Oesophagus	7	7	0
Lymph node	5	5	0
Mediastinum	4	4	0
Liver	1	1	0
Tumour size (mm), median (IQR)	23 (15–49)	20 (11–37)	25 (20–27)
Tumour character, solid/cystic (n)	104/53	92/46	12/7

F, female; FT, fellowship trainee; M, male; VT, visiting trainee.

**Figure 2:**
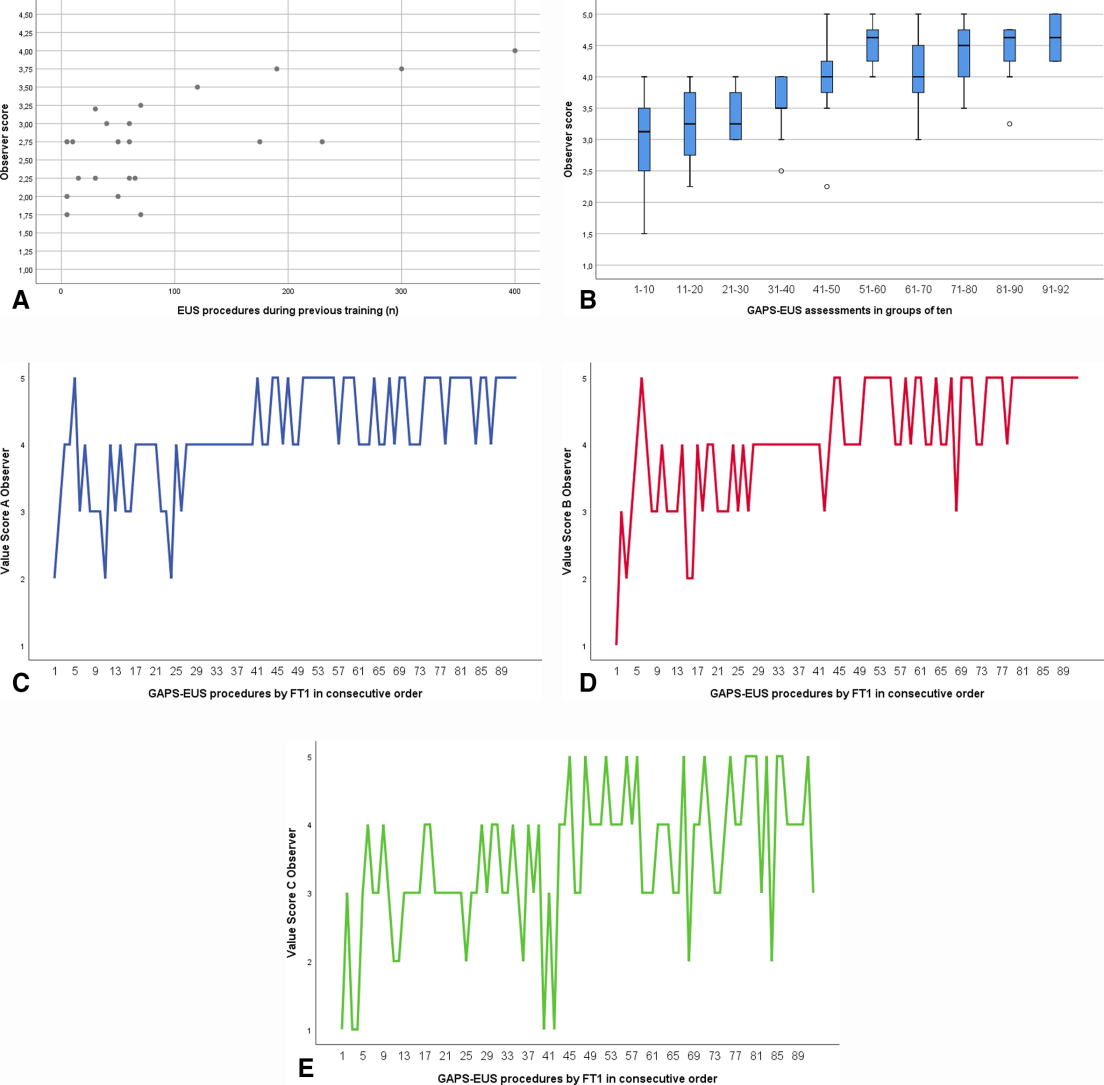
(A) The Observer Score of the first GAPS-EUS assessment in the 3 FTs and in the 19 visiting trainees in relation to the EUS experience among these 22 trainees as measured as the total number of EUS examinations previously performed (under supervision or independently). (B) A bar chart showing the Observer Score of the 92 GAPS-EUS assessments in FT1 in consecutive order and in groups of 10. The error bars symbolise the 95% CIs. (C–E) Three line diagrams showing Observer Score A (C, blue line), Observer Score B (D, red line), and Observer Score C (E, green line) in the 92 consecutive GAPS-EUS assessments of FT1. EUS, endosonography; FT, fellowship trainee; GAPS-EUS, Global Assessment of Performance and Skills in EUS.

The Observer Score of the first GAPS-EUS procedure in each individual trainee (n=22) is plotted in relation to the trainee EUS experience in figure 2A.

### Feasibility and patient safety of GAPS-EUS

The completion rate of the GAPS-EUS assessment tool was 157/157 (100 %).

Potential adverse events related to trainee-performed EUS were recorded in two study patients (gastroenteritis n=1, fever n=1), giving an adverse event rate of 2/157 (1.3 %), both Clavien-Dindo grade I. In no case was hospital submission required, and both patients recovered spontaneously within a few days.

### Reliability of GAPS-EUS

#### Measurement reliability

The measurement reliability (internal consistency) of the GAPS-EUS assessment tool was high both regarding the observer version of the tool (Cronbach’s alpha coefficient=0.87) and the trainee version of the tool (Cronbach’s alpha coefficient=0.89).

#### Inter-rater reliability

The inter-rater reliability of GAPS-EUS, that is, the consistency of assessments in between the observer and the trainee, showed a high correlation (r=0.83, r^2^=0.69, p<0.001).

#### Reliability across the rating scale

**Figure 3:**
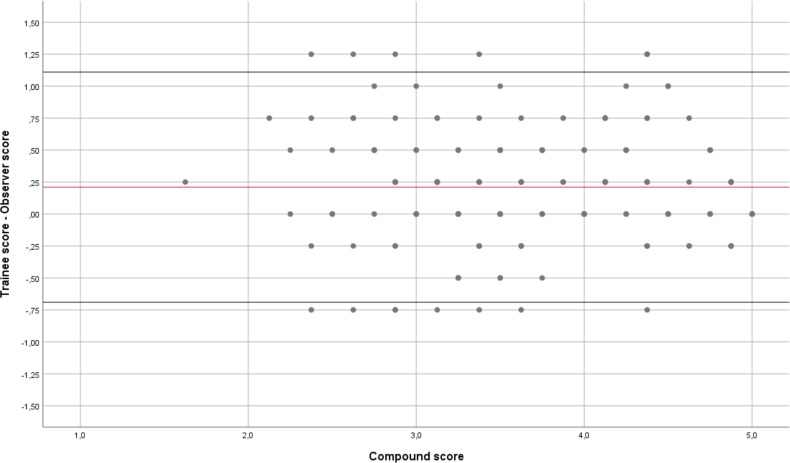
Bland-Altman plot showing the difference of Trainee Score–Observer Score on the Y-axis, including the mean value of all scores as a red line, and the Compound Score (mean of Trainee Score and Observer Score) on the X-axis. The upper and the lower black lines depict the +2 SD and the −2 SD.

The reliability of GAPS-EUS across the rating scale was according to the Bland-Altman plot (figure 3), showing a similar distribution of values in low-score procedures and in high-score procedures. The same plot also shows a tendency that the trainees score their own performance higher as compared with the observers, that is, a positive value of the red line in figure 3.

### Validity of GAPS-EUS

#### Construct validity

The Observer Score in consecutive GAPS-EUS assessments of FT1, that is, the EUS learning curve, is presented in figure 2B. The slope of the curve is positive, indicating progress in EUS skills over time. Meanwhile, there was some discrepancy in between the three key performance indicators (Observer Scores A, B, and C) with a larger score variability and a longer learning curve regarding the assessment of the ultrasound pathology (figure 2E), as compared with echoendoscope handling (figure 2C), and recognition of the ultrasound anatomy (figure 2D).

The mean number of previous EUS procedures performed by the trainees was significantly lower in low performance-level procedures (n=25) compared with high performance-level procedures (n=53) (68 (95% CI 48 to 88) vs 128 (95% CI 116 to 140), p<0.001).

The mean Observer Score in pancreatic lesions (n=79) was significantly lower than the mean Observer Score in non-pancreatic lesions (n=78) (3.35 (95% CI 3.19 to 3.51) vs 3.74 (95% CI 3.58 to 3.90), p=0.002). The mean Observer Score in cystic lesions was significantly lower than the mean Observer Score in solid lesions (3.35 (95% CI 3.16 to 3.54) vs 3.64 (95% CI 3.49 to 3.79), p=0.03).

#### Content validity

In EUS-FNA/EUS-FNB sampling procedures performed by FTs (n=81), the mean score of the sampling manoeuvre (score D) was significantly lower than the mean Observer Score (3.07 (95% CI 2.91 to 3.23) vs 3.51 (95% CI 3.37 to 3.65), p<0.001).

#### External validity

Also in the Italian study site (Bologna), the inter-rater reliability of GAPS-EUS, that is, the consistency of assessments in between the observer and the trainee, showed a high correlation (r=0.78, r^2^=0.61, p<0.001). The mean Observer Score of the initial 27 GAPS-EUS procedures was comparable in the two FTs with similar EUS experience (VT1 (Gothenburg): 3.08 vs VT3 (Bologna): 3.12, p=0.73).

## Discussion

In the current study performed in two European countries, we present and evaluate a new tool (GAPS-EUS) for the assessment of skills and level of performance among trainees in EUS. GAPS-EUS was found easy and safe to use in a real-world, clinical setting, with a recorded high test reliability and validity. By using the GAPS-EUS tool, we could also confirm that certain lesions, such as pancreatic neoplasms, appear difficult from a learning point of view and probably require intense EUS training. Finally, and importantly, the performance level varies significantly in between trainees, which motivates the use of assessment tools like GAPS-EUS.

Not surprisingly, unsupervised self-learning of EUS has been found non-effective, and such training comes with serious concerns regarding procedure quality.^7–9^ Instead, well-organised EUS training programmes result in a high adherence to EUS quality indicators among trainees during their independent practice of EUS after a completed trainee fellowship.^17^ There are suggested assessment tools elaborated for the evaluation of trainee skills and performance in routine endoscopy.^6 18^ However, in many aspects, EUS is a more complex procedure than routine endoscopy. Therefore, unique assessment tools dedicated for EUS have been warranted.^2^

Individuals are variously susceptible to teaching and all learn at their individual pace. We confirmed in the present work that trainees with a similar amount of previous training performed at quite different levels. This result goes in line with findings in previous publications.^17^ Given the unreliable association between the number of training procedures previously performed and the level of performance, supervisors are indeed encouraged to perform trainee assessment with tools like GAPS-EUS.^3^ Meanwhile, some results of the current work should be carefully interpreted since the VTs participating in the study were performing EUS in an external centre together with a temporary supervisor, which could have an impact on the performance level in individual EUS procedures.

Patient safety is another important aspect in trainee-performed endoscopy procedures. We could show that GAPS-EUS applied in supervised, trainee-performed EUS was patient safe. This finding goes in line with two previous studies, in which serious adverse events were uncommon during trainee-performed EUS (abdominal pain 0.7%, pancreatitis 0.2%, and perforation 0.1%,^13^ and pancreatitis 0.4%, perforation 0.06%, and clinically significant bleeding 0.3%).^17^

The implementation of a certain tool is warranted only if the tool is reliable. We recorded a high measurement reliability of the GAPS-EUS assessment tool both considering the score marked by the supervisor and by the trainee. The Crohnbach’s alpha value of GAPS-EUS was comparably high as the Crohnbach’s alpha values presented for gastroscopy and colonoscopy in the study by Vassilou and colleagues.^15^ Furthermore, we found an adequate inter-rater reliability of GAPS-EUS based on a recorded high correlation (r-value) in between the scores marked by the observer and the trainee. Finally, reliability across the rating scale was demonstrated via a similar distribution of values in low score procedures and in high score procedures (figure 3). Further and quite interestingly, the two latter measurements showed that trainees are somewhat prone to overestimate their own skills and performance as compared with their more experienced supervisors. This phenomenon, also known as the Dunning-Kruger effect, is well-known in psychology and it can be identified in a lot of situations in various fields.^19–21^ In short, the Dunning-Kruger effects implicates that, within a certain topic, less experienced individuals are not fully aware of the full complexity of the topic in question. Therefore, they are not aware of the amount of knowledge needed to master all aspects of the topic. Consequently, they regard themselves prematurely as fully skilled and trained. To the best of our knowledge, we are the first to report that the Dunning-Kruger effect is most valid also in advanced endoscopy training. Supervisors should stay alert on this phenomenon during trainee education.

Widespread use of a certain assessment tool requires not only adequate reliability but also a high tool validity. First, we could demonstrate a high construct validity of GAPS-EUS, that is, a tool which is in fact measuring what it is intended to measure, in this specific case, EUS competence. Likely, the acquisition of EUS competence grows and increases gradually over time. As assumed, the design of GAPS-EUS did enable us to depict the learning curve of FT1 with the highest number of procedures performed within the study (n=92). Our results indicate that, rarely a moderate to high performance level can be expected in trainees with a training volume of EUS falling short of 100–150, which is in line with guidelines.^7^

Wani and colleagues are somewhat pioneers in the field of performance assessments in EUS.^3 12 13^ The scoring system used by Wani *et al*^3 12^ was similar to the one used in the current study. However, Wani assessed and scored the trainees in only every 10th EUS; that is, the assessment tool was applied in only 10% of the training procedures. The trainee performance in all other non-assessed procedures cannot be known. Furthermore, there is a potential bias in such a study design since trainees risk to behave and act differently during a procedure if they know that they are being assessed. In our study, no EUS procedures were left out from GAPS-EUS assessment.

Others have investigated the impact of lesion characteristics on the performance of EUS. As in our study, lesions located in the pancreas were found to be more difficult to assess properly.^3 12 13^ A possible explanation is that the size and the echoappearance of the normal pancreas can differ a lot between individuals and between ages. Another explanation might be that previous acute or chronic pancreatitis is not uncommon in patients with pancreatic ductal adenocarcinoma, which makes the assessment demanding. Hence, the pancreas seems to be a challenging organ from a trainee point of view and an organ that requires a long period of training.

Some argue that solid experience in basic EUS is needed before initiating training of EUS-FNA,^22^ while others argue that EUS-FNA can be safely introduced at the start of a trainee fellowship.^23 24^ In the current study, we recorded no serious adverse events related to trainee-performed EUS-FNA. Meanwhile, our findings indicate that a high performance level in other parts of the EUS procedure can be gained faster as compared with EUS-FNA. The mentioned finding also accounts for an adequate content validity of the GAPS-EUS. Too early termination of EUS-FNA training could result in poor sample yield and non-diagnostic cytopathology. Once an acceptable level of trainee competence has been achieved, it might be that it is sufficient for supervisors to assess selected parts of the exam, such as EUS-FNA. Such an approach has been suggested in colonoscopy training.^25^

External validity of GAPS-EUS was supported by using the tool in two separate training centres in Europe, one in Sweden and one in Italy. In both centres, the tool was feasible to use and found patient safe. Moreover, the correlation of the assessment scores marked by the supervisor and the trainee was comparably high in both centres. Furthermore, the assessed level of trainee performance during the initial part of the fellowship period was much alike when comparing two trainees with similar previous experience of EUS. Admittedly, the use and evaluation of GAPS-EUS by yet other institutions involved in EUS training would be beneficial to fully prove external validity.

It remains to be determined what Observer Score and GAPS-EUS grading to be regarded as the cut-off level for sufficiently high competence in EUS and when to consider a trainee as fully trained. We suggest that an Observer Score of at least four in multiple, consecutive GAPS-EUS assessments could be a reasonable threshold. This level of competence implicates that the trainee is capable of mastering most aspects of EUS with minimal support from the supervisor. In any case and to reassure adequate competence, it could be wise to perform some few GAPS-EUS assessments of a previous trainee also a couple of months after completed training.

To the best of our knowledge, the presented work is the first study performed in Europe on the assessment of endosonographers in training. Two endosonographers with a long experience of EUS were engaged as supervisors assessing the trainees. The study is also strengthened by the fact that the trainee assessments were performed both as spot assessments and as repeated assessments over time. Moreover, GAPS-EUS was evaluated in two tertiary endoscopy centres, which should guarantee the validity of the tool. Finally, the study patients were carefully monitored after EUS with no case lost from follow-up.

Admittedly, the current study has some weaknesses. First, and for reasons related to the fellowship positions and the COVID-19 disease, not all three FTs assessed repeatedly could be evaluated for as long as intended. Consequently, two of these three trainees had not yet reached the level of full EUS competence when the repeated assessments had to be terminated. The advantage of evaluating an assessment tool in more than one centre is also a potential pitfall since the tradition in teaching and supervision might differ in between centres and countries. That circumstance needs to be taken into consideration when interpreting the current study. In any case, and as mentioned previously, external validity of the presented results should be strengthened if GAPS-EUS could be tested and evaluated in yet other EUS units. Finally, in the current work, the yield of trainee-performed EUS-FNA/EUS-FNB passes was not separated from the yield of supervisor-performed EUS-FNA/EUS-FNB. Consequently, the evaluation of trainee-performed EUS-FNA/EUS-FNB (score D) was entirely an assessment of the sampling procedure during EUS and did not include aspects on the quality of EUS-FNA/EUS-FNB yield.

In conclusion, GAPS-EUS seems to be an easy and valuable tool for the assessment of skills and level of performance among trainees in EUS. The results of the study also stress the need for assessments of trainees both in early and later stages of training. Without such assessments, there is a risk that poor quality performance is overseen and that trainees with the need for continued training and supervision are not correctly identified.

## Data Availability

Data are available upon reasonable request.
