# Supplementary material for: GAPS-EUS: a new and reliable tool for the assessment of basic skills and performance in EUS among endosonography trainees
Source: BMJ Open Gastroenterol. 2021 Jun 9;8(1):e000660. doi: 10.1136/bmjgast-2021-000660 (PMC8191616; doi:10.1136/bmjgast-2021-000660)
Supplement: Supplementary data [file bmjgast-2021-000660supp001.pdf]

GAPS-EUS SCORESHEET **OBSERVER**

A. Echoendoscope handling and navigation

5. Expertly able to handle scope, intubate, and navigate in all areas and regions of interest

4.

3. Need verbal guidance to intubate, navigate in some areas

2.

1. Unable to intubate or navigate despite coaching

B. Visualization/Recognition of the ultrasound anatomy (trainee to denote organs projected on the monitor)

5. Expertly able to visualize and recognize all organs and structures

4.

3. Need verbal guidance to visualize and recognize some organs or structures

2.

1. Unable to visualize and recognize some organs despite coaching

C. Detection/Assessment of the ultrasound pathology (trainee to describe pathology projected on the monitor)

5. Expertly able to detect, assess and denote the lesion(s) of interest

4.

3. Need additional information and hints to detect, assess and denote the lesion(s) of interest

2.

1. Unable to detect, assess and denote the lesion(s) of interest despite coaching

D. Targeting/Sampling lesions (incl scope positioning, choice of needle, performance of puncture and dialogue with assistants)

5. Expertly able to provide a safe and stable access to a lesion and target it with EUS-FNA/B

4.

3. Need verbal guidance to provide a safe and stable access to a lesion and target it with EUS-FNA/B

2.

1. Unable to provide a safe and stable access to a lesion and target it with EUS-FNA/B despite coaching

E. Quality of examination (includes patient management, efficiency of examination and quality of overall performance)

5. Expertly completes the examination correctly, efficiently and patient friendly

4.

3. Need verbal guidance to perform some steps

2.

1. Could not perform a satisfactory examination despite verbal and manual assistance requiring take over

GAPS-EUS SCORESHEET **OBSERVER**

F. Observer´s assessment of diagnosis (should **NOT** be verbally expressed by observer before asessment by TRAINEE)

Diagnosis:____

[ ] Malignant lesion **[ ] Benign lesion**
